# Selective serotonin 5-HT_1A_ receptor biased agonists elicitdistinct brain activation patterns: a pharmacoMRI study

**DOI:** 10.1038/srep26633

**Published:** 2016-05-23

**Authors:** G. Becker, R. Bolbos, N. Costes, J. Redouté, A. Newman-Tancredi, L. Zimmer

**Affiliations:** 1 Université Claude Bernard Lyon 1, Centre de Recherche en Neurosciences de Lyon, CNRS, INSERM, Lyon, France; 2 Hospices Civils de Lyon, Lyon, France; 3 CERMEP - Imagerie du vivant, Lyon, France; 4 Neurolixis Inc., Dana Point, CA, USA

## Abstract

Serotonin 1A (5-HT_1A_) receptors are involved in several physiological and pathological processes and constitute therefore an important therapeutic target. The recent pharmacological concept of biased agonism asserts that highly selective agonists can preferentially direct receptor signaling to specific intracellular responses, opening the possibility of drugs targeting a receptor subtype in specific brain regions. The present study brings additional support to this concept thanks to functional magnetic resonance imaging (7 Tesla-fMRI) in anaesthetized rats. Three 5-HT_1A_ receptor agonists (8-OH-DPAT, F13714 and F15599) and one 5-HT_1A_ receptor antagonist (MPPF) were compared in terms of influence on the brain blood oxygen level-dependent (BOLD) signal. Our study revealed for the first time contrasting BOLD signal patterns of biased agonists in comparison to a classical agonist and a silent antagonist. By providing functional information on the influence of pharmacological activation of 5-HT_1A_ receptors in specific brain regions, this neuroimaging approach, translatable to the clinic, promises to be useful in exploring the new concept of biased agonism in neuropsychopharmacology.

5-HT_1A_ receptors belong to the family of serotonin receptors, composed of 13 receptor subtypes. 5-HT_1A_ receptors are known to play a key role in serotonin neurotransmission due to their localization both as pre-synaptic receptors located on serotonin cell bodies in the raphe nuclei (somatodendritic receptors) and as post-synaptic heteroreceptors in forebrain areas that receive serotonergic projections. In such areas, 5-HT_1A_ receptors are located on pyramidal and GABAergic neurons of the neocortex and limbic system^1^,^2^. Because of their distribution pattern and of their central role in the modulation of the serotoninergic neurotransmission, 5-HT_1A_ receptors are involved in several physiological and pathological processes and constitute therefore an important therapeutic target for psychiatric^3^ and, more recently, for neurological disorders^4^.

Indeed, it is now well establish that 5-HT_1A_ receptors are one of the main targets for the treatment of mood disorders^5^, with different actions that depend strongly on their localization. For example, activation of somatodendritic receptors by serotonin or 5-HT_1A_ receptor agonists decreases the firing of serotonin neurons in the raphe, and, consequently decreases its terminal release^6^. This decrease is thought to be partially responsible for the delay in onset of the therapeutic action of selective serotonin reuptake inhibitors (SSRI) antidepressants^5^. A recent study renewed this concept, showing that expression levels of 5-HT_1A_ somatodendritic receptors, are critically important for SSRI treatment response by controlling serotoninergic tone^7^. On the other hand, the activation of postsynaptic 5-HT_1A_ receptors seems to be equally important for response to antidepressants^5^,^8^. In a different therapeutic area, 5-HT_1A_ receptor agonism is also known as an important feature of some atypical antipsychotics including clozapine, aripiprazole, ziprasidone and quetiapine^9^,^10^,^11^,^12^.

Recently, 5-HT_1A_ receptors have attracted renewed interest as possible targets in neuropharmacology. For example, it was described that blockade of post-synaptic 5-HT_1A_ receptors, located on pyramidal cells, can improve cognition by enhancing glutamatergic transmission^13^. This led to clinical trials using the 5-HT_1A_ antagonist lecozotan as a procognitive drug in Alzheimer’s disease^14^,^15^. Other 5-HT_1A_ receptor ligands such as sarizotan, buspirone and tandospirone were shown to alleviate dyskinesia in Parkinson’s disease patients^16^,^17^. More recently, it has been reported that 5-HT_1A_ agonists are able to correct breathing dysfunction in mouse models of Rett syndrome opening new perspectives for treatments of this serious orphan disorder^18^.

Although some clinical results with older drugs acting at 5-HT_1A_ receptors were disappointing, all these data suggested that appropriate targeting of 5-HT_1A_ receptors could improve a wide range of CNS disorders if suitable pharmacotherapeutics were available. However, previously-characterized drugs targeting 5-HT_1A_ receptors do not exhibit an ideal profile, firstly, because of their poor selectivity with respect to other targets and, secondly, because of their lack of differentiation between the diverse sub-populations of 5-HT_1A_ receptors that are expressed in different brain regions. The need for ligands that specifically target sub-populations of 5-HT_1A_ receptors encouraged the search for more selective ligands, leading to the identification of novel biased agonists at this target^19^.

Classical agonists of G protein-coupled receptors family (GPCRs), like 5-HT_1A_ receptors, activate G proteins promoting the generation of second messengers such as cyclic adenosine monophosphate (cAMP), calcium, or phosphoinositides. Each GPCR possesses multiple transducing pathways that can elicit desirable (therapeutic) pharmacological effects or unwanted side effects^20^,^21^. The recent concept of “biased agonism” asserts that highly selective agonists can preferentially direct receptor signaling to specific intracellular responses^22^. 5-HT_1A_ receptors are known to interact with Gα_i3_ in dorsal raphe, Gα_o_ and Gα_i3_ in cortex, and Gα_o_, Gα_i1_, Gα_i3_ and Gα_z_ in hypothalamus^23^. Besides this regional heterogeneity in G-subtype proteins coupling, another 5-HT_1A_ receptor ‘downstream’ signaling response, the phosphorylation of extracellular signal-regulated kinase (ERK1/2), also exhibits brain region-specificity^24^. This opens the possibility of identifying drugs that target 5-HT_1A_ receptors in specific brain regions and may therefore exhibit superior therapeutic profiles.

In this context, we characterized novel 5-HT_1A_ agonists, namely F15599 and F13714, which have recently been identified as biased agonists^19^,^25^. These ligands preferentially target cortical heteroreceptors or raphe nuclei somatodendritic autoreceptors, respectively^26^,^27^,^28^. Although their activity has been investigated in tests of neurochemical effects, immediate early gene expression, electrophysiology and behaviour, the differential influence of the compounds on brain region activation (i.e., functional agonism) has not previously been explored using *in vivo* brain imaging techniques. In this study, therefore, we provide, for the first time, *in vivo* functional data of specific effects of biased agonists using magnetic resonance imaging (MRI). Specifically, the activity of rat serotoninergic networks was investigated by a pharmacoMRI (phMRI) approach, in which 5-HT_1A_ receptors were activated by acute pharmacological challenge with biased agonists. We aimed at detecting specific spatiotemporal patterns of brain activity induced either by F15599 (also known as NLX-101) or F13714, in comparison to the prototypical agonist, 8-OH-DPAT and the 5-HT_1A_ receptor antagonist MPPF.

## Results

### Temporal profiles of non-corrected overall BOLD signal changes

**Figure 1:**
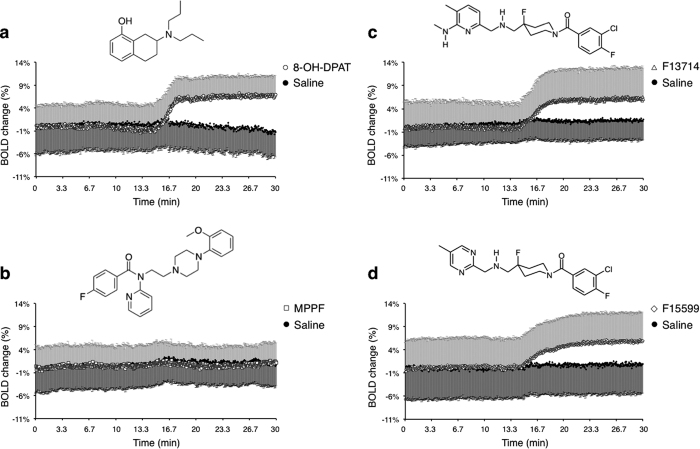
Mean ± SEM change (%) in overall BOLD signal intensity compared to the baseline level. The time courses obtained from each rat, i.e. the average of all pixels from dorsal striatum ROI in individual datasets, were normalized by subtracting the average baseline (zero on average, by definition). Pharmacological challenges (mean + SEM) were carried out 72 hours after the control test, i.e. saline solution (mean–SEM). Upon each time course is drawn the chemical structure of the corresponding compound. (**a**) 8-OH-DPAT (n = 8). (**b**) MPPF (n = 6). (**c**) F13714 (n = 9). (**d**) F15599 (n = 6).

The baseline curves were set on zero by definition, because of the normalization of the non-corrected overall BOLD signal. We found no significant difference in the BOLD signal after MPPF injection in comparison to the corresponding control injection (*i.e*. saline solution). Following injection of the 5-HT_1A_ receptor agonists, we found a significant difference in comparison with the control conditions. Indeed, as shown in Fig. 1, there was a clear increase in the BOLD signal of the dorsal striatum (+7%, +6% and +6% of the basal level for 8-OH-DPAT, F13714 and F15599, respectively). These BOLD signal curves indicated that the drugs elicited overall BOLD effects which can then be analysed using procedures suited to detecting responses at a detailed neuroanatomical level, using suitable correction methods. In fact, the overall BOLD signal is not corrected for statistical significance of the neurovascular effects, over time and by comparing particular brain areas with other regions: only BOLD activation maps, as described below, can be used to compare the BOLD effects between molecules and brain regions.

### BOLD activation maps

**Figure 2:**
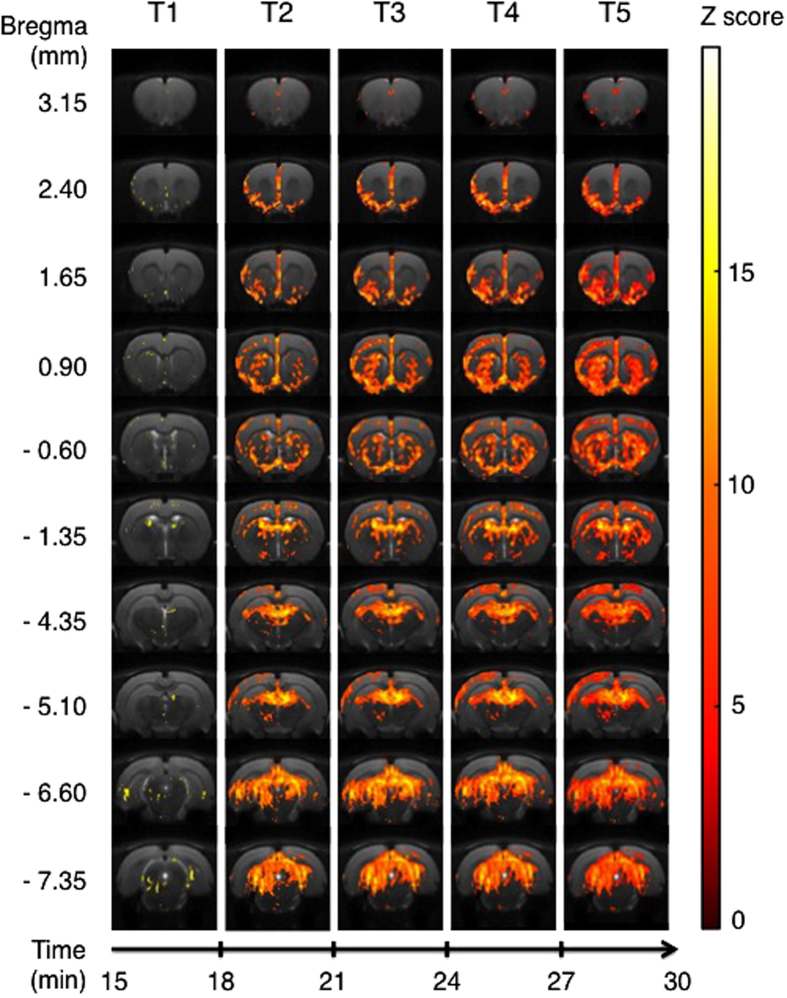
Areas of significant activation following i.p. injection of 8-OH-DPAT. Data are processed in successive 3-min blocks from onset of injection (T1) to the end of acquisition (T5). Statistically significant areas for each time bin, for 8 rats, using an ANOVA for 8-OH-DPAT minus saline injection (p < 0.001 uncorrected). Z score in colour scale, bregma coordinates on the left scale, time scale start at T1 (15 minutes after the beginning of the MRI session).

**Figure 3:**
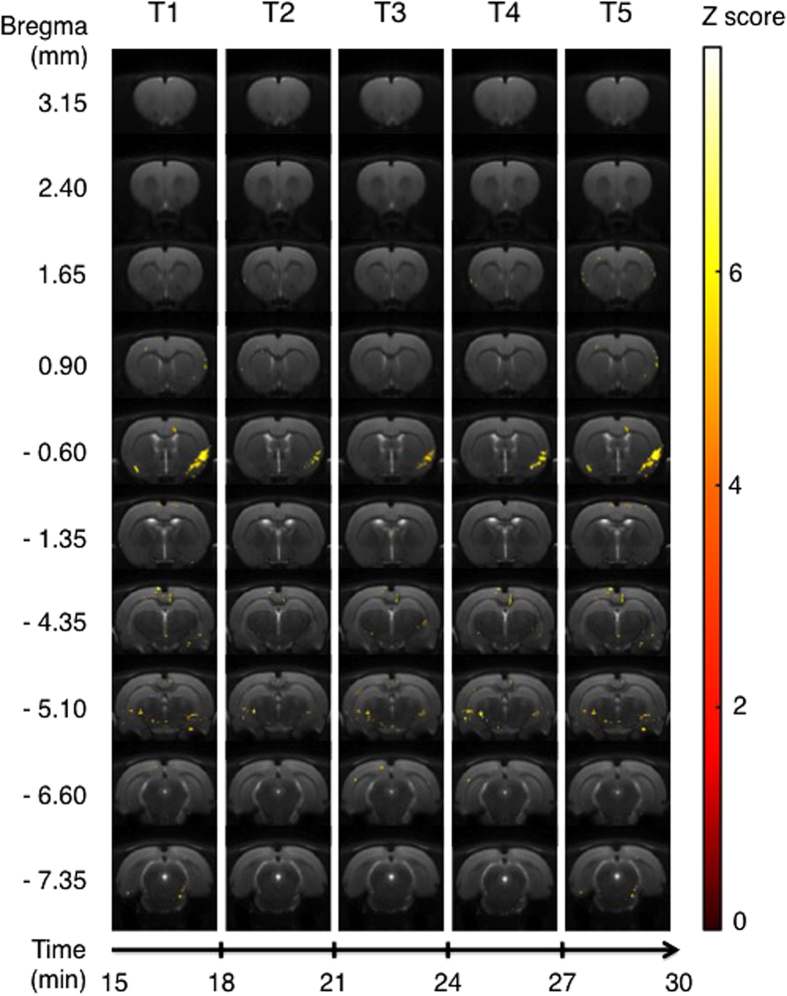
Areas of significant activation following i.p. injection of MPPF. Data are processed in successive 3-min blocks from onset of injection (T1) to the end of acquisition (T5). Statistically significant areas for each time bin, for 6 rats, using an ANOVA for MPPF minus saline injection (p < 0.001 uncorrected). Z score in color scale, bregma coordinates on the left scale, time scale start at T1 (15 minutes after the beginning of the MRI session).

**Figure 4:**
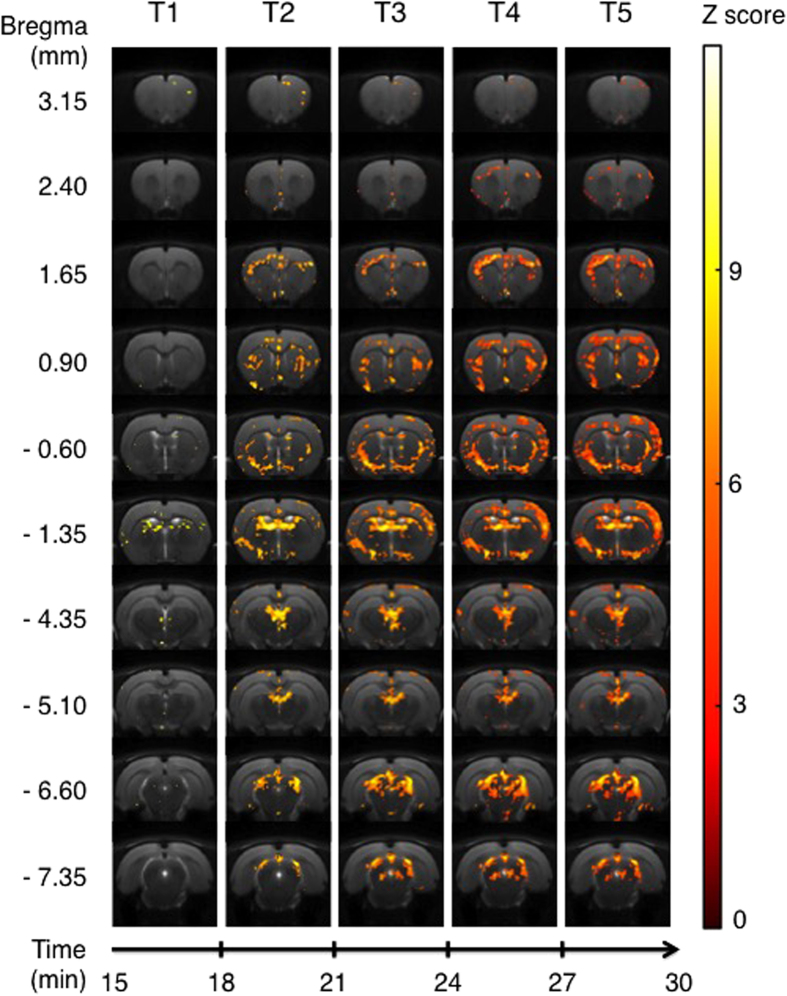
Areas of significant activation following i.p. injection of F13714. Data are processed in successive 3-min blocks from onset of injection (T1) to the end of acquisition (T5). Statistically significant areas for each time bin, for 9 rats, using an ANOVA for F13714 minus saline injection (p < 0.001 uncorrected). Z score in color scale, bregma coordinates on the left scale, time scale start at T1 (15 minutes after the beginning of the MRI session).

**Figure 5:**
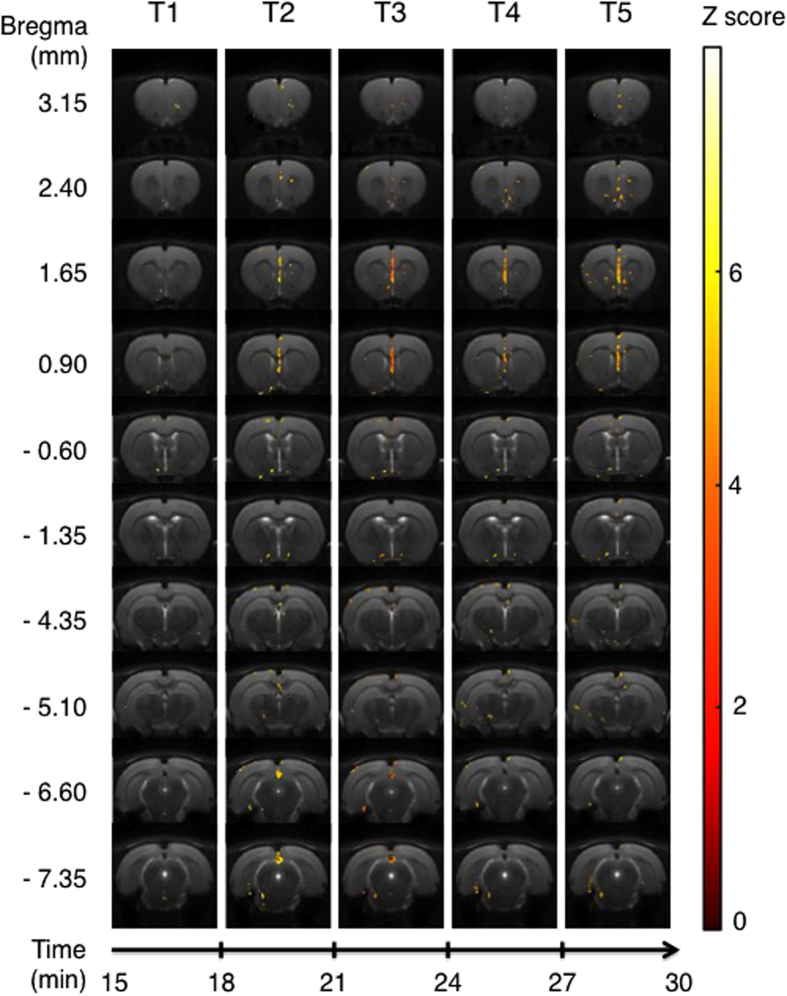
Areas of significant activation following i.p. injection of F15599. Data are processed in successive 3-min blocks from onset of injection (T1) to the end of acquisition (T5). Statistically significant areas for each time bin, for 6 rats, using an ANOVA for F15599 minus saline injection (p < 0.001 uncorrected). Z score in color scale, bregma coordinates on the left scale, time scale start at T1 (15 minutes after the beginning of the MRI session).

The comparison of activation maps generated by treatment with a 5-HT_1A_ ligand minus activation maps generated by treatment with saline was calculated for each time bin [(Tn − T0)_molecule_ − (Tn − T0)_saline_]. The results of these comparisons are shown in Figs 2, 3, 4, 5, which show only those pixels with significant difference between the condition with an agonist injection *versus* the condition with the saline solution injection.

### 8-OH-DPAT

Numerous areas showed a progressive activation, starting from the more rostral slice, at bregma 3.15, which revealed activated voxels in the cingulate cortex from T2 to T5 (Fig. 2). Others voxels were activated, primarily at T4 and T5, in the insular cortex (dorsal and ventral parts) and in the primary motor cortex. The next slice (bregma 2.40) displayed clusters of activated voxels in the entire prefrontal cortex (prelimbic and infralimbic cortices) starting from T2, as well as in the cingulate and motor cortices. The activation was still present in the insular cortex and reached dorsally the primary somatosensory cortex, and ventrally the orbital cortex and the piriform layer, the medial forebrain bundle and the olfactory tubercle. The next slice (bregma 1.65) included the same activated areas, and introduced striatal activation at T4 and T5. At bregma 0.90 the BOLD activation spread to the striatum according to a ventro-dorsal progression. Activation starts in the ventral pallidum and olfactory tubercle at T2, to reach progressively the entire caudate-Putamen at T5. Interestingly, the activation does not include the accumbens nuclei (core and shell). The activation located in the medial septum nucleus and the septo-hippocampus nuclei persisted until T5. The cortical activation started in the piriform layers and reached the somatosensory cortex. The motor and cingulate cortices were also involved starting from T2 and reach a maximum at T5. In the fifth slice, at bregma −0.60, the activation pattern followed the same progression patterns. Activation was seen in the islands of Calleja, preoptic areas, medial forebrain bundle and olfactory tubercle, and then extended to the striatum. Caudate-putamen is first involved, and the activation progresses to the globus pallidus at T5. Cortical activations were also seen in sagittal and cingulate parts. In the medial slice of the brain, at bregma −1.35, the activation started at T2 in thalamic nuclei. Striatal structures started to be involved at T2, and reached a maximal activation at T5. Somatosensory, motor and cingulate cortices were more and more involved from T2 to T5. It is noteworthy that preoptic nuclei are still activated. At bregma −4.35, activated voxels were seen in the dentate gyrus, but also in dorsal nuclei of the thalamus. This activation extended ventrally to thalamic area from T2 to T5. There were also activated voxels in cortical areas, mainly at T4 and T5. In the adjacent slice, at bregma −5.10, activated areas are consistent with those identified in the previous section, and followed a similar temporal pattern. The next slice, (*i.e*. bregma −6.60) had at T2 activated voxels in a part of the dentate gyrus, the subiculum and in posterior cortical areas. This activation spread dramatically to all layers of superior colliculus and to the deep mesencephalic nuclei until T5. The last slice, at bregma −7.35, revealed a considerable activation in the subiculum and post-subiculum, but also in colliculus layers as well as in deep mesencephalic areas from T2 to T5.

### MPPF

BOLD signal was significantly enhanced in only a small area of the piriform and insular cortices in the slice bregma −0.60, from T1 to T5 (Fig. 3). We also noted sparse voxels activated in the cingular cortex at bregma −4.35 and the hippocampus at bregma −5.10.

### F13714

In the case of F13714 minus saline contrast, the activation pattern is shown in Fig. 4. The first slice (bregma 3.15) exhibited only few activated voxels in cortical areas including cingulate, motor and insular cortices. The second slice (bregma 2.40) revealed a cortical activation at T4 and T5, mainly in the motor and cingulate cortices, despite a small cluster in the infralimbic cortex. This cortical activation increased in the following slice (bregma 1.65) involving the cingulate, motor, primary somatosensory and insular cortices. Moreover, there are some activated voxels in septal nuclei. The slice at bregma 0.90 showed, from T2 to T5, small areas activated in the lateral parts of the caudate-putamen, but also in the cingular and motor cortices and in the olfactory tract. There were also activated voxels in the septal nucleus from T2 to T5, as well as in the lateral parts of the cortex. Activation areas in the slice corresponding to bregma −0.60 were firstly ventral, including preoptic areas, anterior amygdaloid areas and ventral pallidum. Starting from T3, activation also involved cortical areas, mainly somatosensory, motor and cingulate cortices. The more extensive activation caused by the F13714 was in the slice at bregma −1.35. At that level, the main activation was observed in thalamic nuclei, starting from T1, and subsequently extended ventrally. Ventral nuclei were activated from T2 to T5, including preoptic and amygdaloid nuclei. This slice showed also a sharp cortical activation, in all cortical areas except piriform layers. At the medial level (bregma −4.35), activated areas were observed in the dentate gyrus, the median habenular nuclei and the paraventricular thalamic nuclei. Cortical activated voxels were still detectable from T3 to T5. While this cortical activation was reduced at the level of bregma −5.10, from T3 to T5, the most significant activation was seen in the subiculum and reached the superior colliculus. In the last two slices, at level bregma −6.60 and −7.35, the activation remained in the subiculum and in the post-subiculum, respectively, from T2 to T5. At bregma −6.60, it progressively expanded to layers of the colliculi, while at −7.35, the others activated areas were located in periaqueductal grey matter.

### F15599

We noted, for the F15599 minus saline test, an extremely specific and limited activation (Fig. 5). The first rostral slice showed activated voxels in the optical cortex at T1 and T2, and some others in pre- and infralimbic cortices mainly at T5. The next slice (bregma 2.40) confirmed this activation while T4 and T5 time bins displayed more activated voxels in septo-hippocampal nuclei. The cingulate cortex was clearly activated from T2 to T5 in the slices at bregma 1.65 and 0.90, as were some voxels in median and septo-hippocampal nuclei. This slice also showed activated voxels in the ventral part, probably corresponding to the olfactory tract. At the coordinate bregma −0.60 and −1.35, those preoptic nuclei were still activated. Cortical activation was observed mainly in several parts of the retrosplenial cortex (slices −6.60 and −7.35).

### Summary of the results

These results show, for the first time, distinct BOLD activation patterns between two 5-HT_1A_ “biased agonists”, a prototypical 5-HT_1A_ agonist and a “silent” 5-HT_1A_ antagonist. In the case of F13714 (0.04 mg/kg), activation involved a circuit composed of thalamo-cortical areas, ventral preoptic and pallidum nuclei in its caudal part. In the median brain, only a small part of the hippocampus (*i.e*. subiculum) was activated, and then the colliculus and mesencephalic nuclei. BOLD stimulation induced by F15599 (0.16 mg/kg) was strictly restricted to a network composed by the limbic and retrosplenial cortices, and the median septum.

**Figure 6:**
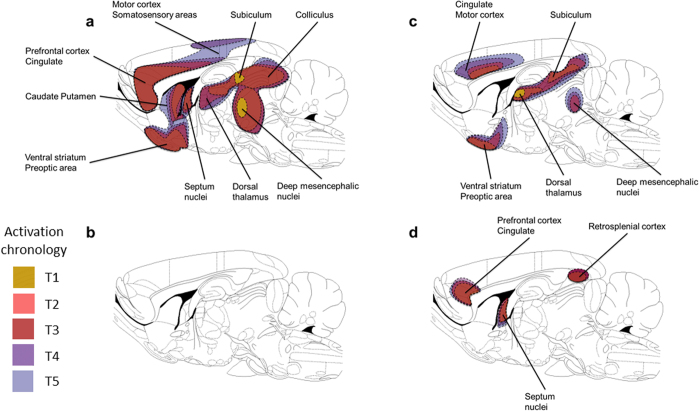
Topography of the main activated regions after injection of 5-HT_1A_ ligands. **(a)** 8-OH-DPAT as a prototypical 5-HT_1A_ agonist. (**b**) MPPF as a 5-HT_1A_ “silent antagonist” (without any activation). (**c**) F13714 as a 5-HT_1A_ biased agonist. (**d**) F15599 as a 5-HT_1A_ biased agonist. Colours indicate the chronology of activation (yellow, T1; orange, T2; red, T3; purple, T4; blue, T5).

On the contrary, 8-OH-DPAT (0.32 mg/kg), induced a widespread activation across the whole brain, including both ventral and dorsal striatum, medio-rostral hippocampus, the dorsal thalamus along the midline, and cortical areas (*i.e*. limbic, motor and somatosensory). The rostral part of the midbrain was also deeply activated. In sharp contrast, blockade of 5-HT_1A_ receptors by the selective antagonist, MPPF (0.16 mg/kg), didn’t lead to any specific BOLD activation except for small cortical areas. Figure 6 summarizes the topography of these main networks.

## Discussion

To our knowledge, this is the first *in vivo* imaging contribution to the pharmacological concept of biased agonism. We choose to study the influence of biased agonists on functional magnetic resonance imaging (fMRI) by detecting with a reduced time-resolution the blood oxygen level dependent (BOLD) signal. The application of fMRI methods to examine the central effects of pharmacological agents used as stimuli drugs has been dubbed as pharmacological-MRI (phMRI)^29^. Briefly, the method relies on the detection of a weak NMR signal enhancement (around 2 to 5%) due to the decrease in concentration of deoxyhemoglobin, that is paramagnetic, within the activated brain zones, translating an increase of oxygen consumption. The phMRI approach therefore offers an *in vivo* whole-brain view on resulting changes in brain activity and is a non-invasive method to map spatio-temporal changes in neuronal activities under acute pharmacological challenge. Indeed, phMRI is a useful tool to study serotonin neurotransmission, for example, in animal models^30^, as well in humans in a translational manner, as suggested by the increased number of phMRI studies focused on 5-HT-modifying drugs^31^,^32^.

In the present study, pharmacological actions of the biased agonists F15599 and F13714, used at appropriate doses, led to the activation of very different neuronal networks.

Before discussing the results and the perspectives they open, methodological considerations about experimental procedures, data processing and interpretations should be explained. Although our study doesn’t aim to elucidate the phenomena underlying neurovascular coupling^33^ (and particularly receptor-mediated hemodynamic modifications), the present protocol was designed to achieve maximal reproducibility of BOLD signal measurement. First, each rat was its own control by fMRI acquisition under saline solution injection (placebo condition), three days before the pharmacological challenge with one of the 5-HT_1A_ ligands. The maintenance of strictly comparable conditions between control and test image acquisitions allows us to avoid bias due to potential stimulation of brain regions due to the venous injection of the drug samples. In any case, we made efforts to minimize the influence of the injections by normalizing the volume at 1ml/200g of body weight and by monitoring body temperature and respiration rates to ensure that there was no change in these parameters during acquisition and between the two conditions. It is important to mention that the use of anaesthesia, itself a neuropharmacological manipulation, requires careful consideration in phMRI experiments. Isoflurane is widely used in fMRI because it produces a stable anaesthesia over prolonged periods of time, with minimal physiological modifications^34^. Neuronal coupling can be detected at up to 2% isoflurane^35^, and functional connectivity remains accessible under isoflurane anaesthesia, although it is reduced compared to that in conscious animals^36^.

Another crucial issue was the choice of the doses of each 5-HT_1A_ receptor ligand. In all cases, the tested doses of the 5-HT_1A_ agonists were below those that are known to elicit hypothermia for these compounds^37^. Indeed, doses were chosen on the basis of tests reflecting behavioural and neurochemical 5-HT_1A_ receptor targeting *in vivo*, and this at their minimal doses. For F15599, the dose of 0.16 mg/kg is known to preferentially activate cortical 5-HT_1A_ receptors in microdialysis experiments, with only modest activation of 5-HT_1A_ autoreceptors^28^. This is associated with beneficial properties on cognitive tests, reversing PCP-induced working and reference memory deficits^38^,^39^. Conversely, F13714 potently and preferentially activates 5-HT_1A_ autoreceptors at the dose of 0.04 mg/kg^24^,^26^, an effect that is associated with memory effects (see below) and also produces potent anti-dyskinetic activity in rodent tests of L-DOPA-induced abnormal involuntary movements^40^. 8-OH-DPAT activates 5-HT_1A_ auto and heteroreceptors at doses at or below 0.64 mg/kg^41^ so a dose of 0.32 mg/kg was selected here. It is important to note that, at the chosen doses, the effects of F15599 and F13714 are completely abolished by co-administration with a selective 5-HT_1A_ receptor antagonist (see cited references) indicating that their actions are specifically mediated by 5-HT_1A_ receptors, consistent with their known exceptional selectivity for this target *in vitro*^27^,^42^.

Some debate remains as to whether this is true for 8-OH-DPAT. Indeed, as discussed below, this prototypical 5-HT_1A_ agonist is known to interact with 5-HT_7_ receptors at modest doses and, possibly, 5-HT reuptake sites, in addition to 5-HT_1A_ receptors^43^. In comparison, the antagonist, MPPF, has previously been shown to block 5-HT_1A_ receptors *in vivo* at the chosen dose of 0.16 mg/kg^44^,^45^.

Having carefully addressed the methodological challenges of the fMRI procedure, we observed, for the first time, differential neuronal activation maps corresponding to 5-HT_1A_ biased agonism effects. Despite targeting the same 5-HT_1A_ receptor family, each biased agonist, F15599 and F13714, elicited a BOLD effect propagating to different cortical and subcortical regions, consistent with their distinct targeting of 5-HT_1A_ receptor subpopulations reported previously (see discussion below). In contrast, the BOLD activations patterns of F15599 and F13714 were strikingly different to those obtained with 8-OH-DPAT, a prototypical 5-HT_1A_ agonist, widely used in neuropharmacology. Our results revealed that 8-OH-DPAT activation spread notably in hippocampus and thalamus. We can interpret this large activation by the fact that 8-OH-DPAT is a mixed 5-HT_1A_/5-HT_7_ agonist^46^,^47^. The diffuse BOLD activation in caudal areas may be due to the presence of 5-HT_7_ receptors in these regions^47^, while strong activation in striatum could be explained by dopamine release induced by activation of postsynaptic 5-HT_1A_ receptors and by direct action of 8-OH-DPAT itself on 5-HT_7_ receptors. Moreover, the 8-OH-DPAT activation maps we obtained agree with a previous phMRI study with 5-HT_7_ receptor ligand reporting a similar BOLD activation in prefrontal cortex, striatum and thalamic areas^48^.

As negative pharmacological control, we used MPPF, a structural analogue of the silent antagonist WAY-100635^45^. It is noteworthy that MPPF injection, at the same dose as F15599, had just a slight cortical effect, but did not activate any of the above areas, even at the latest time of acquisition:these results therefore support the silent antagonist profile of MPPF. Nevertheless, these data should be interpreted with caution, given that Canese *et al*. observed BOLD activation under antagonist challenge^48^. Although it is not possible to make a direct comparison with the study by Canese *et al*., due to the different MPPF concentrations used, it remains possible that a higher dose of MPPF could produce BOLD activation.

F15599, the first biased agonist we used, is a highly selective and efficacious 5-HT_1A_ agonist in a variety of signal transduction models^27^. F15599 has a strong preferential activity on cortical 5-HT_1A_ receptors, probably mediated through Gα_i_ and pERK1/2 signalling pathways^28^,^37^,^49^. Such F15599 postsynaptic preference translates to behavioural manifestations, e.g. an attenuation of PCP-produced deficits of memory/cognition at the same dose we used herein^39^. Our *in vivo* data revealing activation of cortical networks by F15599 are in accordance with this “procognitive” profile.

In contrast, F13714, which also has high affinity and selectivity for 5-HT_1A_ receptors (Ki, 0.05 nM)^50^, preferentially activates somatodendritic 5-HT_1A_ receptors and impairs cognitive performance in rat at low doses similar to the ones used here^26^,^39^,^51^. The present imaging data suggest that F13714’s impact on memory may be related to the activation of a hippocampo-striatal neuronal network. Indeed, activation of hippocampal 5-HT_1A_ receptors is known to induce memory deficits, at least in the case of 8-OH-DPAT^52^. However, the present data demonstrating marked BOLD signal in striatal regions by F13714 may also provide a basis for this agonist’s potent anti-dyskinetic effects, recently reported in a rat model of Parkinson’s disease^40^.

At a neurochemical level, it may be speculated that the differential effects of F15599 and F13714 observed here probably involve regulation of multiple neuronal populations. For example, both GABAergic and glutamatergic neurons are likely to be involved in the cortical effects of F15599 observed by Llado-Pelfort *et al*.^28^ and regulation of serotonergic neurons and of dopamine release likely underlies the effects of F13714^26^,^40^.

Overall, it appears clear that, at the present doses, biased agonists that selectively target the same receptor subtype can exhibit widely divergent brain BOLD patterns, presumably because of distinct targeting of signal transduction pathways in different brain regions, which, in turn, can have specific haemodynamic effects. Such observations may have extensive implications at both a basic science level, for increased understanding of the role of different brain regions, and at a therapeutic level, for the development of improved drug treatments that target the appropriate brain regions involved in the brain disorder of interest. For example, the present data suggest that serotonergic drugs eliciting a BOLD signal in specific areas of the frontal cortex may be attractive candidates for treatment of cognitive dysfunction. In contrast, drugs targeting cortico-striatal networks-striatal networks may constitute effective anti-dyskinetic pharmacotherapies.

## Conclusions

These present data reveal, for the first time by *in vivo* imaging, the specific pattern of activation of 5-HT_1A_ biased agonists and their difference with a prototypical agonist, at least at the doses tested herein. These data suggest that phMRI imaging could make an important contribution to the *in vivo* characterization of novel biased agonists. Supplementary studies are now scheduled to expand this initial work. Firstly, a longer acquisition time would be interesting to determine the duration of action of the drugs for activation of different brain regions and according to the respective pharmacokinetics of each 5-HT_1A_ ligand. Secondly, increased doses of the drugs will be tested in order to explore the extent of the dose-dependency of biased-agonist effects. Thirdly, by associating the present data with PET imaging of receptor occupancy, it should be possible to determine a correlation of the present BOLD effects with the level of 5-HT_1A_ receptor drug-occupancy of each ligand. Finally, and thanks to the translational aspect of fMRI, this paradigm can be transferred to larger animals and, ultimately to human subjects, opening a new way in the investigation of biased agonists as drug candidates.

## Methods

### Animals and experimental procedures

Twenty-nine male Sprague-Dawley adult rats (Charles River laboratories, France) of 277.8 ± 19.6 g (mean at the beginning of the protocol) were used within all the experiments. The animals were hosted in standard temperature and humidity conditions with a 12h/12h light/dark cycle. Food and water were provided *ad libitum*. All experiments were performed in accordance with European guidelines for care of laboratory animals (2010/63/EU) and were approved by the Animal Use Ethics Committee of the University of Lyon (Université Claude Bernard Lyon 1).

### Experimental procedures

Anesthesia was performed using an approved system (TEM Sega, Lormont, France). First, the animals were placed in an induction box and a mixture of 4% isoflurane (Laboratoire Bekamont, Boulogne Billancourt, France) and air with 30% oxygen was delivered at 1L/min flow rate.

A catheterization procedure was performed for an intra-peritoneal injection of pharmacological agents during the MRI acquisition. The catheter was maintained by ligation to the abdominal wall and a one meter-long tube was connected to carry out pharmacological agents or saline injections.

The animals were placed on prone position in a dedicated plastic holder (Bruker Biospec Animal Handling Systems, Germany), adapted with a stereotactic system allowing animal’s head immobilization. The anaesthesia was delivered via a dedicated cone mask and maintained at 2% of isoflurane during the entire MRI session. The body temperature was maintained at 37 ± 0.2 °C by means of a temperature-controlled water circuit integrated in the dedicated holder. A respiratory sensor was also placed on the animal’s abdomen allowing a continuous monitoring of the respiration rate (Trigger ECG Unit RH V.0, Rapid Biomedical, Germany).

### MRI Protocol

The MRI protocol was carried out on a 7-Tesla Bruker Biospec MR system (Bruker Biospin GbmH, Germany) equipped with a 400 mT/m maximal amplitude gradient set and controlled by a workstation interfaced with ParaVision5.1 software for data acquisition and post-processing (Bruker, Germany). A transmitting body coil (outer diameter, 112mm and inner diameter, 72mm) and a receive-only surface coil (25 mm of diameter) were used for rat brain image acquisitions. A 2D anatomical T2-RARE image (Rapid Acquisition with Relaxation Enhancement) was obtained with the following parameters: echo time (TE): 69.1 ms, repetition time (TR): 5000 ms, field of view: 3 × 1.5 cm^2^, matrix 256 × 128 pixels, spatial resolution: 117 μm^2^, RARE factor: 8, acquisition time: 4 minutes. Ten contiguous slices of 1.5-mm thickness were acquired, covering the whole rat brain.

To measure local cerebral hemodynamic variations during pharmacological stimulations, the BOLD (Blood-Oxygen-Level-Dependent) functional MRI method was employed. A T2* (Echo Planar Imaging) sequence was used with the following parameters: TE/TR: 25/3000 ms, matrix 128 × 64 pixels, spatial resolution: 234 μm^2^. Ten slices were acquired with the identical geometry of the anatomical T2-RARE scan that facilitated further region of interest (ROI) definition. Each BOLD fMRI session consisted in one series of 600 repetitions (scanning time of 30 minutes).

All animals were scanned under two distinct conditions: a control condition, which consisted of saline solution (NaCl 0.9%) injection, and, 72 hours later, a second challenge condition consisting of a pharmacological molecule injection. The 29 animals were randomly divided into 4 groups, one for each pharmacological molecule used. The molecules were following 5-HT_1A_ ligands: 8-OH-DPAT (n = 8), F13714 (n = 9), F15599 (n = 6) and MPPF (n = 6). The injected doses were 0.32 mg/kg for 8-OH-DPAT, 0.04 mg/kg for F13714, 0.16 mg/kg for F15599, and 0.16 mg/kg for MPPF. The injected volumes of the molecule and saline solutions were calibrated at 0.5 mL per 100g of body weight. The pharmacological or saline solutions were injected 15 min after the beginning of the 30 min BOLD fMRI session, resulting in 300 baseline scans and 300 post-injection scans. The injections were carried out over a period of 30 seconds, followed by a 600 μL saline flush.

## Data Analysis

### Regional time course responses

Time courses of the BOLD signal intensities were examined in the dorsal striata of each animal. The dorsal striata were manually delineated on T2 slices according to a rat brain atlas^53^ and this for each rat of the protocol. Then, the time courses of the BOLD signal were extracted. Temporal points were obtained by averaging the BOLD signal intensity values through all pixels within the ROI. For each individual and anatomical ROI, baseline time points were averaged. This mean value was used to normalize the regional BOLD time course.

### BOLD activation maps and statistics

Data were analyzed using Statistical Parametric Mapping software (SPM8, The Wellcome Trust Center for Neuroimaging, London, UK). Three preprocessing steps were performed: (1) images realignment using a spatial cross-correlation algorithm to correct possible head movements during acquisition, (2) spatial normalization using a standardized MRI template^54^, and (3) spatial smoothing using an isotropic Gaussian filter [1 × 1 × 1 mm]. All these preprocessing steps enabled inter-subject averaging.

**Figure 7:**
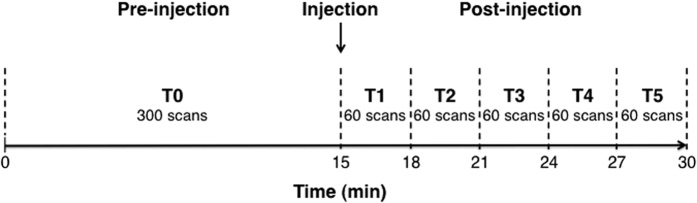
phMRI protocol time line. The 30-min scan was divided into two 15-min sections (pre-injection and post-injection). T0 was the pre-injection scan and included all 300 volumes in the 15-min time bin. The injection scans were divided into five lots of 60 scans time bins (30 min each, T1 to T5).

For each group, a first-level analysis (intra-subject) was performed both for saline and 5-HT_1A_ molecule conditions. The 600 scans per session were divided into six time bins (Fig. 7), as suggested by McKie *et al*.^55^. The 300 baseline scans (15 min) were defined as the first time bin (T0). The 300 post-injection scans (15 min) were divided into 5 time bins of 60 scans each (T1 to T5). All six time bins were then introduced into a SPM block design using a General Linear Model (GLM) approach.

For both conditions (saline and molecule) of each subject, the five post-injection time bins (T1 to T5) were individually compared to T0 using a student *t*-test (*p* < 0.01). This first-level analysis resulted in five first-level parametric contrast images per subject and per condition, corresponding to contrasts [Tn − T0]_condition_.

Second-level (random effect) analyses were carried out to determine whether these individual contrast images statistically increase in a significant way at a voxel level between time bins (T0, T1, T2, T3, T4) for each molecule. This second-level used analysis of variance (ANOVA) with the times bins contrasts as between-group factor (molecule versus saline injections) for each time bin. This second-level ANOVA was computed for each group (8-OH-DPAT; MPPF; F13714; F15599) resulting in activation maps for each molecule and each time bin [(Tn − T0)_molecule_ − (Tn − T0)_saline_]. A significant threshold was set up at *p* < 0.001 uncorrected.

## Additional Information

**How to cite this article**: Becker, G. *et al*. Selective serotonin 5-HT_1A_ receptor biased agonists elicit distinct brain activation patterns: a pharmacoMRI study. *Sci. Rep*. **6**, 26633; doi: 10.1038/srep26633 (2016).
